# Excisions of severe cervical dysplasia: Are there mandatory diameters of the cone that need to be considered?

**DOI:** 10.4274/jtgga.2017.0036

**Published:** 2017-12-15

**Authors:** Daniel A. Beyer, Achim Rody, Natalie Schmidt, Christoph Cirkel, Kay Neumann

**Affiliations:** 1 Westpfalz-Klinikum GmbH, Kaiserslautern, Germany; 2 Department of Obstetrics and Gynaecology, Schleswig-Holstein University, Campus Luebeck, Luebeck, Germany

**Keywords:** Dysplasia, high-grade squamous intraepithelial lesion, loop electrosurgical excision procedures, preterm delivery, depth

## Abstract

**Objective:**

To achieve optimal depth for negative margin cones after loop electrosurgical excision procedures (LEEP) for cervical dysplasia.

**Material and Methods:**

Retrospective cohort analysis of LEEP cones of 201 patients with cervical dysplasia during a four-year period. Analysed cones were divided into two different groups: cones with negative margins without dysplasia, and cones with margins positive for dysplasia. In order to determine the cut-off value of the depth of the resected cones, receiver operating characteristic (ROC) analysis was performed.

**Results:**

Negative margins were found in 71.0% (n=49) of all cones, whereas positive margins were reported in 29.0% (n=20). Negative margin cones were achieved in 100% with a cone depth of ≥20 mm. A resection depth between 10-19.9 mm led to 73.0% negative margin cones. Calculation of cone volume shows for 2.0 cm3, a sensitivity of 79% and a specificity of 64%. Statistical analysis using an ROC model showed p=0.002.

**Conclusion:**

Forth greatest safety of patients, cone depths from LEEPs for cervical dysplasia should be ≥20 mm to achieve negative margins.

## INTRODUCTION

More than 270.000 women die per year from cervical cancer according to the World Health Organization (1). Thus, prevention and early detection of cervical carcinoma and its dysplastic precursor lesions are of essential importance. Precursor lesions of cervical carcinoma can be classified as low- or high-grade squamous intraepithelial lesions (LSIL or HSIL). These lesions are located around the transformation zone of the cervix uteri. Before acquiring the ability for malignant invasion, precursor lesions may rest for up to ten years (2). Grade of dysplasia and time of its detection determine the chance of invasive growth (3).

Preinvasive lesions of the cervix uteri and cervical cancer are still a major health issue in Germany.

The treatment of dysplastic lesions of the cervix uteri is challenging; in Germany the majority of patients is older than 30 years according to public health statistics (4); therefore, the question of family planning arises when discussing excisions of the cervix uteri. On the one hand, excisions of dysplastic lesions of the cervix uteri have to demonstrate negative margins (i.e., without dysplasia) to maximize safety for patients. On the other hand, Noehr et al. (5) described an increasing risk for preterm delivery depending on the depths of the resected cone. Due of this dilemma, there are no clear recommendations for depth of excisions of dysplastic lesions of the cervix uteri (6, 7, 8). Thus, the aim of this study was to find the optimal size of resected cone in order to achieve negative margin samples.

## MATERIAL AND METHODS

**Figure 1:**
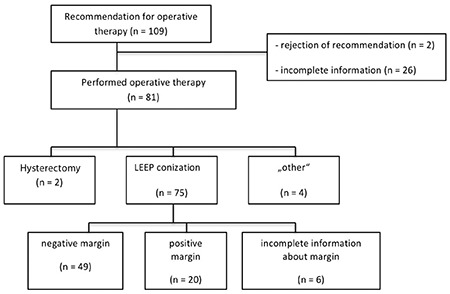
Flowchart of patients

This study was approved by the ethics board of the University of Luebeck, Germany (registration number 12-234). The Department of Obstetrics and Gynecology at University Hospital of Luebeck is certified and registered for treating patients with dysplasia according to national guidelines by the “Arbeitsgemeinschaft Zervixpathologie und Kolposkopie e.V.”. Data of all patients (n=517) who were referred to the Department of Obstetrics and Gynecology because of cervical dysplasia during a period of four years were screened retrospectively, as depicted in Figure 1.

Inclusion criteria were defined as: recommendation for operative therapy, current Papanicolaou smear test, histologic confirmed cervical dysplasia, and available standardized histologic analysis report.

Exclusion criteria were defined as: pregnancy ≥16 weeks of gestation, negative informed consent. LEEPs were performed under colposcopic surveillance using a Leisegang 3MVS LED colposcope (45.000-52.000 Lux; 300 mm free working distance) with integrated camera. Portio-examinations: natively, aceto acid 5% and iodine stained. All LEEPs were performed in a sterile operation room using tungsten snare electrodes (ERBE) of different sizes under regional or general anaesthesia. Endocervical curettage was routinely performed prior to LEEP. Hemorrhage was stopped using high-voltage spray for cauterization. No sutures were used. The resected cone was marked with a suture at twelve o´clock for orientation and transported to the pathology department in a formalin container. The examination of the cone was perfomed according to Westra et al. (9).

The cone volume was calculated using the formula based on the data of the acquired pathologic report.

Surgical margins were considered positive when the dysplastic lesion was closer than 1 mm to the margin, otherwise for >1 mm distance to the lesion it was considered negative.

For better analysis, resected cones were differentiated into three different categories according their resection depth: plane: 0-9.9 mm, medium: 10-19.9 mm, deep: 20-100 mm.

### Power calculation

A statistical a priori-power analysis was performed for sample size estimation, based on the assumption that the effect size in this study was medium using Cohen’s (1988) criteria. Thus, sample size of 109 observations would achieve 80% power.

### Statistical analysis

Data were collected in Microsoft® Access 2003 from MS Office® Package. Statistical analysis was performed using IBM SPSS Statistics® version 22.0 for windows. Analysis included the Mann-Whitney U test for continuous data, the chi-square test for categorical data, and Fisher’s exact T-test. The level of significance was defined α=0.05%. For statistical evaluation of the negative margin samples according to the volume of the cone, the area under the curve of a receiver operating characteristic analysis (ROC) was calculated.

## RESULTS

In total, 109 patient received recommendations for operative therapy and were included in this study, as shown in Figure 1. The mean age of the patients was 31.2 years with a standard deviation of 7.3 years. The basic demography of the study population is shown in Table 1.

### Margins of cones from LEEPS

**Figure 2:**
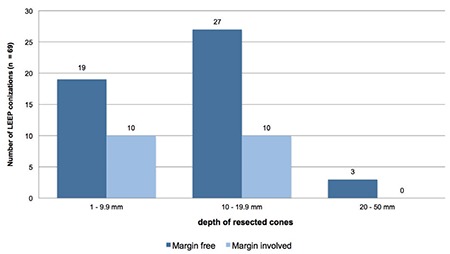
Depth of resected cones and margin status

Sixty-nine excisions from LEEPs could be analyzed: Negative margins were found in 71.0% (n=49) of all LEEPs. A positive margin was reported by the pathologist in 29.0% (n=20) of the cases. Negative margin cones were achieved in 100% of cases for a depth equal or larger than 20 mm (n=3). A resection depth of 10-19.9 mm led to a negative margin rate of 73.0% (n=37) (Figure 2). The histologic results of LEEPs are depicted in Table 2.

### Receiver operating characteristic analysis

**Figure 3:**
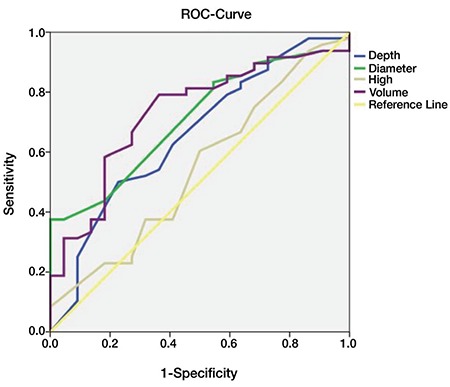
ROC analysis

Initially, the volume of the cone was analyzed. A resected volume of 2.0 cm3 displayed a sensitivity of 79% with a specificity of 64%. A ROC model displayed a statistical significance of p=0.002 for margin status. According to the depth of the resected cone, a statistical significance of 0.036 was found. A depth of ≥19 mm revealed a sensitivity of 79% with a specificity of 41%, which is reflected in Figure 3 (for legend for Figure 3, Table 3).

## DISCUSSION

In this study, the histologic status of the margins of resected cones correlated clearly with the depth of the resected cone. Best safety was achieved for a depth equal or larger than 20 mm.

Previous studies investigating resection depth and margin status found conflicting results. Some studies found optimal depth for 10 mm resections (7), or confirmed 20 mm as the optimal depth (6), whereas the study of Öz et al. (6) found no correlation between resection depth and margin status. The use of cold-knife excisions might have influenced the results of the studies of Öz et al. (6) and Kliemann et al. (7), which makes a comparison with the results of the present study difficult.

For positive margins of cones from LEEPs, a previous meta-analysis showed a relative risk of 6.1 for recurrence of cervical dysplasia (CIN 2/3), which stresses the importance of negative margins of LEEP samples (10). Therefore, from this point of view, LEEPs should aim at great depth of cones (>20 mm) to maximize safety for patients.

On the other hand, Noehr et al. (5) described an estimated 6% increase in risk for preterm delivery with every additional millimeter of excised cervical tissue. Because the average age for family plannung has risen it is becoming increasingly important to resect only as much tissues as necessary to achieve a negative margin.

The results of the current study suggest a depth ≥20 mm for best safety. However, the estimated odds ratio (OR) for preterm delivery is already significantly elevated (OR ≈1.5) for a depth of ≈13 mm and is increasing to around OR ≈3.0 for depths around 20 mm (4). This illustrates the dilemma for surgeons and emphasizes the need for detailed patient education about risk of positive margins versus risk of preterm delivery in a latter pregnancy.

In conclusion, this study suggests a resection depth of ≥20 mm to achieve histologic negative margins of cones from LEEPs for cervical dysplasia. On the other hand, patients need to be educated about the increased risk for preterm delivery that comes with a greater depth.

### Study limitations

The retrospective design and low number of patients with positive margins (n=20) limit this study. Following the recommendation of operative therapy, 2 patients refused surgery and 26 patients had insufficient data about demography or on the histologic report (n=6), which could create a selection bias.

## Figures and Tables

**Table 1:**
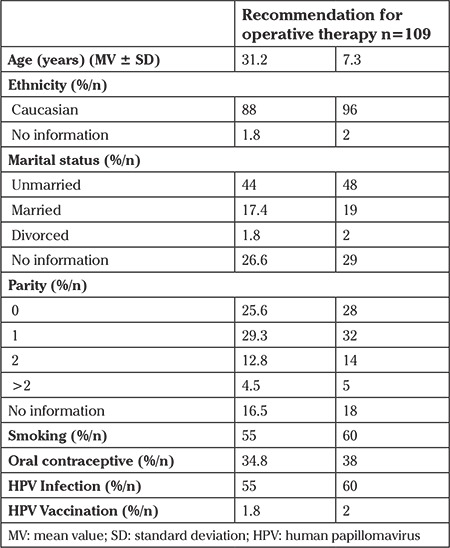
Demography of study population

**Table 2:**
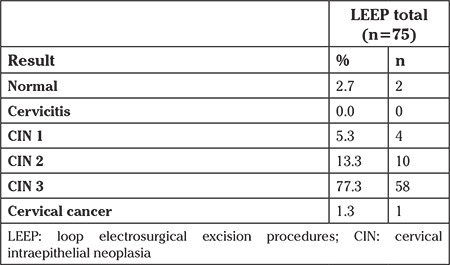
Results of LEEPs

**Table 3:**
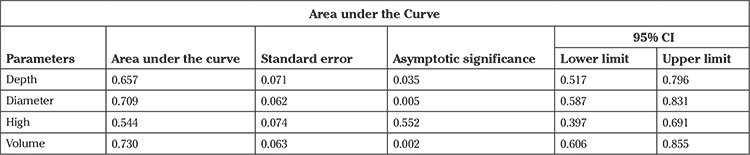
Legend for ROC analysis
